# Bosonic Representation of Matrices and Angular Momentum Probabilistic Representation of Cyclic States

**DOI:** 10.3390/e25121628

**Published:** 2023-12-06

**Authors:** Julio A. López-Saldívar, Olga V. Man’ko, Margarita A. Man’ko, Vladimir I. Man’ko

**Affiliations:** 1 Instituto de Ciencias Nucleares, Universidad Nacional Autonoma de Mexico, Apdo. Postal 70-543, Ciudad de Mexico 04510, Mexico; 2 Department of Theoretical Physics, Moscow Institute of Physics and Technology, Dolgoprudnyi, Moscow 141700, Russia; manko@sci.lebedev.ru; 3 Russian Quantum Center, Skolkovo, Moscow 143025, Russia; 4 Laboratory of Quantum Information Technologies, National University of Science and Technology (MISIS), Moscow 119049, Russia; 5 Lebedev Physical Institute, Leninskii Prospect 53, Moscow 119991, Russia; mankoov@lebedev.ru (O.V.M.); mankoma@lebedev.ru (M.A.M.); 6 Special Educational Scientific Center, Bauman Moscow State Technical University, The 2nd Baumanskaya Street 5, Moscow 105005, Russia

**Keywords:** tomographic representation, Jordan-Schwinger map, Lie algebras, coherent states, angular momentum, continuous variable systems

## Abstract

The Jordan–Schwinger map allows us to go from a matrix representation of any arbitrary Lie algebra to an oscillator (bosonic) representation. We show that any Lie algebra can be considered for this map by expressing the algebra generators in terms of the oscillator creation and annihilation operators acting in the Hilbert space of quantum oscillator states. Then, to describe quantum states in the probability representation of quantum oscillator states, we express their density operators in terms of conditional probability distributions (symplectic tomograms) or Husimi-like probability distributions. We illustrate this general scheme by examples of qubit states (spin-1/2 su(2)-group states) and even and odd Schrödinger cat states related to the other representation of su(2)-algebra (spin-*j* representation). The two-mode coherent-state superpositions associated with cyclic groups are studied, using the Jordan–Schwinger map. This map allows us to visualize and compare different properties of the mentioned states. For this, the su(2) coherent states for different angular momenta *j* are used to define a Husimi-like *Q* representation. Some properties of these states are explicitly presented for the cyclic groups $C_{2}$ and $C_{3}$. Also, their use in quantum information and computing is mentioned.

## 1. Introduction

The development of modern technologies is based on the discovery of quantum mechanics, in which the states of particles are described by wave functions (pure states) and density matrices [1,2] (mixed states), which are quite different from the states in classical mechanics. Due to this, there was a dream of researchers to understand the notion of quantum states, using a classical concept like probability distributions describing the states. This activity results in the introduction of quasi-probabilities like the Wigner function [3], the Husimi function [4,5], and the Glauber–Sudarshan function [6,7], but the possibility to find the probability description of quantum states was found much later; we use this probability representation of quantum states in our work.

The probability representation of quantum states was constructed in [8,9]. In this representation, an arbitrary state, i.e., the wave function or the density operator [10], as well as the state vector in the Hilbert space, can be invertible mapped onto the probability distribution, which contains the same information on the quantum state as its density operator. Also, as was found in [11,12], for a given set of N×N matrices, including the matrices realizing a representation of Lie algebra, there exists an invertible map of these matrices onto the set of operators acting in the Hilbert space of the bosonic oscillator operators, which are quadrature forms of the oscillator’s creation and annihilation operators, and they realize the representation of the Lie algebra under consideration. This fact provides the possibility to associate conventional probability distributions with an arbitrary Lie algebra.

Thus, our aim was to realize the construction of probability distributions of Lie algebras by means of tomographic probability distributions describing quantum oscillator states or quasi-probability representations as the Husimi function [4] by the use of the Jordan–Schwinger map. The Jordan–Schwinger representation has been used in several studies. For example, it has been used to define representations of Lie groups, i.e., by obtaining the matrix elements of finite and infinitesimal group transformations in the bases of coherent and Fock states [13,14], for unitary groups in the context of molecular, atomic, and nuclear physics [15,16,17,18,19,20,21,22], and for Cayley–Klein groups [23,24,25].

The probabilistic representation of quantum mechanics has been discussed in different works, namely the symplectic tomographic distribution for cyclic states was obtained in [26], the use of the tomographic representation of states was studied in [27], and some aspects of the time evolution of a quantum system in a parametric amplifier in the tomographic representation was performed in [28]. In [29], the classical Universe emerging from the tomographic representation of quantum systems was presented. In [30], the tomographic representation for the Friedmann–Robertson–Walker model within the Loop Quantum Cosmology framework was elaborated. The operator-sum representation of a quantum process was extended to the probability representation of quantum mechanics in [31]. Some relevant developments in classical and quantum tomography were reviewed in [32]. The relation between the tomographic description and the convolution algebra of discrete groupoids on the Hilbert space was discussed in [33]. The embedded manifolds in the space of quantum states by means of a quantizer–dequantizer system and their dynamical invariants were proposed in [34]. The generalizations of the Radon transform and the Weyl–Wigner quantization were described in [35] to discuss some aspects of the tomographic representation. In [36], the tomographic representation was used to discuss the Schrödinger cat experiment. The behavior of two-qubit states subjected to tomographic measurements and a tomographic discord that maximizes the Shannon mutual information were defined in [37]. The Radon transform and its relation to the theory of the metaplectic group and the quadratic Fourier transform were developed in [38]. The notion of polar duality from convex geometry was used to characterize pure Gaussian states in terms of partial information on the covariance ellipsoid [39].

The states used in this work contain the symmetries for the regular *n*-sided polygon in the phase space; in other words, the states are invariant under all the operations contained in the cyclic group $C_{n}$, where *n* denotes the degree of the group [40,41,42,43]. The cyclic group $C_{n}$ contains the rotations, over which the *n*-sided regular polygon is invariant; in other words, $C_{n}=\left\{I,R\left(2\pi /n\right),R\left(4\pi /n\right),\ldots ,R\left(2\left(n-1\right)\pi /n\right)\right\}$. This group has *n* irreducible representations, with characters equal to the roots of unity $\chi_{\lambda ,r}^{\left(n\right)}=e^{i2\pi (\lambda -1)(r-1)/n}$, where λ represents the irreducible representation; λ=1,2,…,n. Then, the group characters have the following orthogonality conditions:

$$\frac{1}{n}\sum_{r=1}^{n}\chi_{\lambda ,r}^{\left(n\right)}\;\chi_{\lambda^{\prime },r}^{(n)*}=\delta_{\lambda ,\lambda^{\prime }}\;\mathrm{and}\;\frac{1}{n}\sum_{\lambda =1}^{n}\chi_{\lambda ,r}^{\left(n\right)}\;\chi_{\lambda ,r^{\prime }}^{(n)*}=\delta_{r,r^{\prime }};$$

this property makes these characters an adequate quantity to define orthogonal quantum states. This definition has been performed, using first the harmonic oscillator coherent states and also general states in the phase space. For the one-dimensional coherent states, this superposition reads

(1) $$|\psi_{n}^{\left(\lambda \right)}\rangle =M_{\lambda }^{\left(n\right)}\sum_{r=1}^{n}\chi_{\lambda ,r}^{\left(n\right)}|\alpha_{r}\rangle ,$$

where $\alpha_{r}=R\left(\frac{2\pi r}{n}\right)\left(\begin{array}{c} \alpha_{R}\\ \alpha_{I} \end{array}\right)$ are the rotations of a parameter $\alpha =\alpha_{R}+i\alpha_{I}$ in the complex space. These superpositions of coherent states have some interesting properties, such as sub-Poissonian photon statistics and negative Wigner functions [42]. Additionally, they form an orthogonal set of states, which can be employed to carry quantum information. In this work, the superpositions of coherent states are bimodal of the form

(2) $$|\psi_{n}^{\left(\lambda \right)}\rangle =\sum_{r=1}^{n}C_{\lambda ,r}|\alpha_{r},\beta_{r}\rangle ,$$

which also form an orthogonal set of states.

This article is organized as follows.

In Section 2, we give a short review of the Jordan–Schwinger map construction for arbitrary Lie algebras and present some examples. In Section 3, we discuss the bosonic representation of the su(2)-group and the connection between the angular momentum and bimodal Fock states, coherent states, and their superpositions. In Section 4, we present the probabilistic representation of cyclic states by means of the Husimi-like distribution and symplectic tomographic representations. Finally, we present conclusions and prospects in Section 5.

## 2. Jordan–Schwinger Map of Lie Algebras

In the present work, we make extensive use of the Jordan–Schwinger map, which allows us to associate angular momentum states with Fock number states. Below, we give a quick summary of the map.

### 2.1. Preliminaries

We make use of sets of N×N matrices $L_{k}$, with k=1,2,…,R, satisfying the commutation relations:

(3) $$\left[L_{k},L_{p}\right]=\sum_{s=1}^{R}f_{kps}L_{s};\;k,p,s=0,1,2,\ldots ,R,$$

where $f_{kps}$ are constant parameters of the system, e.g., the structure constants associated with a unitary group.

Given a set of states of *N*-mode harmonic oscillator $|n_{1},n_{2},\ldots ,n_{N}\rangle$, with the wave function $\psi_{n_{1}n_{2}}\left(x_{1},x_{2}\right)=\langle x_{1}x_{2}|n_{1}n_{2}\rangle$, there exist the creation and annihilation operators:

(4) $${\hat{a}}_{q}=\frac{1}{\sqrt{2}}\left(x_{q}+\frac{\partial }{\partial x_{q}}\right),\;{\hat{a}}_{q}^{†}=\frac{1}{\sqrt{2}}\left(x_{q}-\frac{\partial }{\partial x_{q}}\right),$$

with the commutation relations:

(5) $$\left[{\hat{a}}_{p},{\hat{a}}_{q}\right]=0,\;\left[{\hat{a}}_{p},{\hat{a}}_{q}^{†}\right]=\delta_{qp};\;p,q=1,2,\ldots ,N,\;\left[{\hat{a}}_{p}^{†},{\hat{a}}_{q}^{†}\right]=0.$$

Also, the Hilbert space of the *N*-mode oscillator states has the basis of vectors:

(6) $$|n_{1},n_{2},\ldots ,n_{N}\rangle =\frac{{\left({\hat{a}}_{1}^{†}\right)}^{n_{1}}{\left({\hat{a}}_{2}^{†}\right)}^{n_{2}}\cdots {\left({\hat{a}}_{N}^{†}\right)}^{n_{N}}}{\sqrt{n_{1}!n_{2}!\cdots n_{N}!}}|0,0,\ldots 0\rangle ,$$

where the inner product between different vectors is defined as

(7) $$\langle n_{1},n_{2},\ldots ,n_{N}|n_{1}^{\prime },n_{2}^{\prime },\ldots ,n_{N}^{\prime }\rangle =\delta_{n_{1}n_{1}^{\prime }}\delta_{n_{2}n_{2}^{\prime }}\cdots \delta_{n_{N}n_{N}^{\prime }},$$

and the vacuum state for a two-mode system is described by the following wave function:

(8) $$\Psi_{00}\left(x_{1},x_{2}\right)=\frac{e^{-(x_{1}^{2}+x_{2}^{2})/2}}{\sqrt{\pi }}\;.$$

The tomographic representation of a quantum state Ψ(x) is defined as the following integral:

(9) $$w_{\psi }\left(X|\;\mu ,\nu \right)=\frac{1}{2\pi |\nu |}{\left|\int_{-\infty }^{\infty }\Psi \left(x\right)e^{\frac{i\mu }{2\nu }x^{2}-\frac{iX}{\nu }x}\;dx\right|}^{2};$$

this tomogram is a probability distribution, which fully characterizes a one-dimensional quantum system in a rotated and re-scaled system X=μq+νp; μ=scosθ, $\nu =s^{-1}\sin\theta$. For the bi-dimensional state $\Psi (x_{1},x_{2})$, the generalization is given as follows:

(10) $$w_{\psi }\left(X,Y|\;\mu_{1},\nu_{1},\mu_{2},\nu_{2}\right)=\frac{1}{4\pi^{2}|\nu_{1}\left|\right|\nu_{2}|}{\left|\int_{-\infty }^{\infty }\Psi \left(x_{1},x_{2}\right)e^{\frac{i\mu_{1}}{2\nu_{1}}x_{1}^{2}-\frac{iX}{\nu_{1}}x_{1}+\frac{i\mu_{2}}{2\nu_{2}}x_{2}^{2}-\frac{iY}{\nu_{2}}x_{2}}\;dx_{1}\;dx_{2}\right|}^{2}.$$

The Husimi quasi-probability distribution Q(α) is defined for any state |ψ〉 as the overlap probability between the state |ψ〉 and the harmonic oscillator coherent state $|\alpha \rangle =e^{-{\left|\alpha \right|}^{2}/2}\sum_{n=0}^{\infty }\frac{\alpha^{n}}{\sqrt{n!}}|n\rangle$; it reads

(11) $$Q_{\psi }\left(\alpha \right)=\frac{1}{\pi }{\left|\langle \alpha |\psi \rangle \right|}^{2},$$

where we can deal with optical coherent states or coherent states associated with the su(2) algebra [44]. In the bimodal case, one has

(12) $$Q_{\psi }\left(\alpha ,\beta \right)=\frac{1}{\pi^{2}}{\left|\langle \alpha ,\beta |\psi \rangle \right|}^{2},$$

and with these definitions in mind, we explore the Jordan–Schwinger map for matrices.

### 2.2. Jordan–Schwinger Map of *N*-Dimensional Matrices

We discuss the possibility to use the map of matrices *A*, *B*, and *C* for operators $\hat{A}$, $\hat{B}$, and $\hat{C}$, such that, from the commutation relation of matrices $\left[A,B\right]=C$ follows the commutation relation $\left[\hat{A},\hat{B}\right]=\hat{C}$. This can be performed by employing the properties of the creation and annihilation operators ${\hat{a}}_{i}^{†}$ and ${\hat{a}}_{k}$, which satisfy the bosonic commutation relations $\left[{\hat{a}}_{i},{\hat{a}}_{k}\right]=\left[{\hat{a}}_{i}^{†},{\hat{a}}_{k}^{†}\right]=0$ and $\left[{\hat{a}}_{i},{\hat{a}}_{k}^{†}\right]=\delta_{ik}$. Then, it can be demonstrated that defining the operators $\hat{A}$, $\hat{B}$, and $\hat{C}$ in the following form:

(13) $$\begin{array}{c} \hat{A}=\sum_{i=1}^{N}\sum_{k=1}^{N}A_{ik}\;{\hat{a}}_{i}^{†}{\hat{a}}_{k},\;\hat{B}=\sum_{i=1}^{N}\sum_{k=1}^{N}B_{ik}\;{\hat{a}}_{i}^{†}{\hat{a}}_{k},\;\hat{C}=\sum_{i=1}^{N}\sum_{k=1}^{N}C_{ik}\;{\hat{a}}_{i}^{†}{\hat{a}}_{k} \end{array}$$

yields the desired commutation relation $\left[\hat{A},\hat{B}\right]=\hat{C}$. We point out that this method is of a particular use when working with matrices, which are the generators of an algebraic group as the unitary group or any other group, whose generators can be represented by finite matrices.

As an example of the general procedure, we consider a two-mode quantum oscillator with complex wave function Ψ(x,y) and the creation and annihilation operators:

(14) $$\begin{array}{c} {\hat{a}}_{1}=\frac{1}{\sqrt{2}}\left(x+\frac{\partial }{\partial x}\right),\;{\hat{a}}_{2}=\frac{1}{\sqrt{2}}\left(y+\frac{\partial }{\partial y}\right),\;{\hat{a}}_{1}^{†}=\frac{1}{\sqrt{2}}\left(x-\frac{\partial }{\partial x}\right),\;{\hat{a}}_{2}^{†}=\frac{1}{\sqrt{2}}\left(y-\frac{\partial }{\partial y}\right). \end{array}$$

Then, it can be shown that the excited states ∣1,0〉 and ∣0,1〉 form a basis of the Hilbert space of the qubit, where

(15) $$\Psi_{10}\left(x,y\right)=\frac{\sqrt{2}\;x}{\sqrt{\pi }}\;e^{-x^{2}/2}\;e^{-y^{2}/2},\;\Psi_{01}\left(x,y\right)=\frac{\sqrt{2}\;y}{\sqrt{\pi }}\;e^{-x^{2}/2}\;e^{-y^{2}/2},$$

and satisfy the relations:

(16) $${\hat{a}}_{1}^{†}\;{\hat{a}}_{2}\;\Psi_{10}\left(x,y\right)=0,\;{\hat{a}}_{2}^{†}\;{\hat{a}}_{1}\;\Psi_{01}\left(x,y\right)=0.$$

To show this property, we use the Jordan–Schwinger map previously described for the two-mode oscillator. This means that matrices *A*, *B*, and *C* are chosen as the Pauli matrices $S_{i}=\frac{\sigma_{i}}{2},$ where $\sigma_{x}=\left(\begin{array}{cc} 0 & 1\\ 1 & 0 \end{array}\right)$, $\;\sigma_{y}=\left(\begin{array}{cc} 0 & -i\\ i & 0 \end{array}\right)$, and $\sigma_{z}=\left(\begin{array}{cc} 1 & 0\\ 0 & -1 \end{array}\right).$ Then, after the procedure, we have the operators:

(17) $${\hat{S}}_{x}=\frac{1}{2}\left({\hat{a}}_{1}^{†}\;{\hat{a}}_{2}+{\hat{a}}_{2}^{†}\;{\hat{a}}_{1}\right),\;{\hat{S}}_{y}=\frac{i}{2}\left({\hat{a}}_{2}^{†}\;{\hat{a}}_{1}-{\hat{a}}_{1}^{†}\;{\hat{a}}_{2}\right),\;{\hat{S}}_{z}=\frac{1}{2}\left({\hat{a}}_{1}^{†}\;{\hat{a}}_{1}-{\hat{a}}_{2}^{†}\;{\hat{a}}_{2}\right),$$

which satisfy the commutation relations of the su(2) Lie algebra $\left[{\hat{S}}_{i},{\hat{S}}_{k}\right]=i\;\epsilon_{ikl}\;{\hat{S}}_{l}$. Thus, the states $\Psi_{10}\left(x,y\right)$, $\Psi_{01}\left(x,y\right)$ and their normalized linear combinations realize an irreducible representation of the spin-1/2 symmetry group of the two-mode oscillator.

It was shown [45] that the density matrices of quantum states can be described by the conditional probability distributions expressed in terms of wave functions. Here, we consider these probability distributions for qubit states.

We use the general formula for the symplectic tomogram of the Fock state ∣n〉:

(18) $$w_{n}\left(X|\mu ,\nu \right)=\frac{\exp\left[-X^{2}/\left(\mu^{2}+\nu^{2}\right)\right]}{\sqrt{\pi (\mu^{2}+\nu^{2})}}\cdot \frac{1}{2^{n}n!}{\left|H_{n}\left(\frac{X}{\sqrt{\mu^{2}+\nu^{2}}}\right)\right|}^{2};\;n=0,1,2,\ldots$$

For the qubit states and their number state counterparts, $\left(\begin{array}{c} 1\\ 0 \end{array}\right)⟷|1,0\rangle$ and $\left(\begin{array}{c} 0\\ 1 \end{array}\right)⟷|0,1\rangle$, we obtain symplectic tomograms:

(19) $$w_{10}\left(X,Y|\mu_{1},\nu_{1},\mu_{2},\nu_{2}\right)=\frac{\exp\left[-\left\{X^{2}/\left(\mu_{1}^{2}+\nu_{1}^{2}\right)\right\}-\left\{Y^{2}/\left(\mu_{2}^{2}+\nu_{2}^{2}\right)\right\}\right]}{\sqrt{\pi^{2}\left(\mu_{1}^{2}+\nu_{1}^{2}\right)\left(\mu_{2}^{2}+\nu_{2}^{2}\right)}}\cdot {\left|H_{1}\left(\frac{X}{\sqrt{\mu_{1}^{2}+\nu_{1}^{2}}}\right)\right|}^{2}$$

and

(20) $$w_{01}\left(X,Y|\mu_{1},\nu_{1},\mu_{2},\nu_{2}\right)=\frac{\exp\left[-\left\{X^{2}/\left(\mu_{1}^{2}+\nu_{1}^{2}\right)\right\}-\left\{Y^{2}/\left(\mu_{2}^{2}+\nu_{2}^{2}\right)\right\}\right]}{\sqrt{\pi^{2}\left(\mu_{1}^{2}+\nu_{1}^{2}\right)\left(\mu_{2}^{2}+\nu_{2}^{2}\right)}}\cdot {\left|H_{1}\left(\frac{Y}{\sqrt{\mu_{2}^{2}+\nu_{2}^{2}}}\right)\right|}^{2}.$$

For qubit states in the *x* basis, we have a superposition of states $\frac{1}{\sqrt{2}}\left(\begin{array}{c} 1\\ 1 \end{array}\right)⟷\frac{1}{\sqrt{2}}(|1,0\rangle +\left|0,1\rangle \right)$ and $\frac{1}{\sqrt{2}}\left(\begin{array}{c} 1\\ -1 \end{array}\right)⟷\frac{1}{\sqrt{2}}(|1,0\rangle -\;|0,1\rangle )$, which define tomograms as conditional probability distributions given by

(21) $$\begin{array}{ccc} & & w_{1,\pm 1}\left(X,Y|\mu_{1},\nu_{1},\mu_{2},\nu_{2}\right)=\frac{1}{8\pi^{2}\left|\nu_{1}\nu_{2}\right|}\\ & & \times {\left|\int \left[\Psi_{10}\left(x_{1},x_{2}\right)\pm \Psi_{01}\left(x_{1},x_{2}\right)\right]\cdot \exp\left(\frac{i\mu_{1}}{2\nu_{1}}x_{1}^{2}-\frac{iX}{\nu_{1}}x_{1}+\frac{i\mu_{2}}{2\nu_{2}}x_{2}^{2}-\frac{iY}{\nu_{2}}x_{2}\right)\;dx_{1}\;dx_{2}\right|}^{2}, \end{array}$$

where $\Psi_{10}\left(x_{1},x_{2}\right)$ and $\Psi_{01}\left(x_{1},x_{2}\right)$ are given by Equation (15)

For the qubit state $\left(\begin{array}{c} 1\\ 0 \end{array}\right)⟷|1,0\rangle$, we obtain the symplectic tomogram given by Equation (19), which can be presented explicitly as follows:

(22) $$w_{10}\left(X,Y|\mu_{1},\nu_{1},\mu_{2},\nu_{2}\right)=\frac{2X^{2}}{\pi {\left(\mu_{1}^{2}+\nu_{1}^{2}\right)}^{3/2}{\left(\mu_{2}^{2}+\nu_{2}^{2}\right)}^{1/2}}\exp\left(-\frac{X^{2}}{\mu_{1}^{2}+\nu_{1}^{2}}-\frac{Y^{2}}{\mu_{2}^{2}+\nu_{2}^{2}}\right).$$

For the qubit state $\left(\begin{array}{c} 0\\ 1 \end{array}\right)⟷|0,1\rangle$, the probability distribution describing the qubit state with negative spin projection on the *z* axis can be seen in Equation (20), which also is written in the explicit form as follows:

(23) $$w_{01}\left(X,Y|\mu_{1},\nu_{1},\mu_{2},\nu_{2}\right)=\frac{2Y^{2}}{\pi {\left(\mu_{1}^{2}+\nu_{1}^{2}\right)}^{1/2}{\left(\mu_{2}^{2}+\nu_{2}^{2}\right)}^{3/2}}\exp\left(-\frac{X^{2}}{\mu_{1}^{2}+\nu_{1}^{2}}-\frac{Y^{2}}{\mu_{2}^{2}+\nu_{2}^{2}}\right).$$

The excited oscillator state $|n_{1},n_{2}\rangle$; $n_{1},n_{2}=0,1,2\ldots$ describes the qudit state of spin s=N/2; $N=n_{1}+n_{2}$, where the oscillator wave function in the position representation reads

(24) $$\Psi_{n_{1}\;n_{2}}\left(x_{1},x_{2}\right)=\frac{1}{\sqrt{\pi }}\;\exp\left[-\frac{x_{1}^{2}+x_{2}^{2}}{2}\right]\cdot \frac{1}{\sqrt{2^{n_{1}+n_{2}}\;n_{1}!n_{2}!}}\;H_{n_{1}}\left(x_{1}\right)\cdot H_{n_{2}}\left(x_{2}\right).$$

The approach developed provides the possibility to construct arbitrary qudit states in the tomographic probability representation, where arbitrary qudit states are associated with probability distributions described by Hermite polynomials of two variables with an extra Gaussian factor.

Thus, we associated tomographic probability distributions with qubit states, calculating the integrals of linear combinations of the wave functions belonging to the first excited states of the two-dimensional oscillators. For a qutrit state, the construction of the probability representation on the base of symplectic tomograms means that one has to calculate the integrals with the functions:

(25) $$\Psi \left(x_{1},x_{2}\right)=C_{1}\Psi_{2\;0}\left(x_{1},x_{2}\right)+C_{2}\Psi_{1\;1}\left(x_{1},x_{2}\right)+C_{3}\Psi_{0\;2}\left(x_{1},x_{2}\right).$$

Such integrals provide symplectic tomograms describing qutrit states in the probability representation. An analog of this method was used in [46,47]. The approach can be extended for arbitrary $N=n_{1}+n_{2}$; N=1,2,3… Also, one can apply it for Schrödinger cat states or other algebraic groups. As an example of the bosonic representation, we briefly discuss the case of the su(3) algebra.

### 2.3. Example: Bosonic Representation of su (3) Algebra

In the case of the unitary group and its subgroups, we arrive at the standard definition of the generators in terms of boson operators [15]. To see an example, we make a quick review of the procedure for su(3). The generators of the su(3) algebra are the Gell–Mann matrices:

(26) $$\begin{array}{c} \lambda_{1}=\left(\begin{array}{ccc} 0 & 1 & 0\\ 1 & 0 & 0\\ 0 & 0 & 0 \end{array}\right),\;\lambda_{2}=\left(\begin{array}{ccc} 0 & -i & 0\\ i & 0 & 0\\ 0 & 0 & 0 \end{array}\right),\;\lambda_{3}=\left(\begin{array}{ccc} 1 & 0 & 0\\ 0 & -1 & 0\\ 0 & 0 & 0 \end{array}\right),\;\lambda_{4}=\left(\begin{array}{ccc} 0 & 0 & 1\\ 0 & 0 & 0\\ 1 & 0 & 0 \end{array}\right),\\ \lambda_{5}=\left(\begin{array}{ccc} 0 & 0 & -i\\ 0 & 0 & 0\\ i & 0 & 0 \end{array}\right),\;\lambda_{6}=\left(\begin{array}{ccc} 0 & 0 & 0\\ 0 & 0 & 1\\ 0 & 1 & 0 \end{array}\right),\;\lambda_{7}=\left(\begin{array}{ccc} 0 & 0 & 0\\ 0 & 0 & -i\\ 0 & i & 0 \end{array}\right),\;\lambda_{8}=\frac{1}{\sqrt{3}}\left(\begin{array}{ccc} 1 & 0 & 0\\ 0 & 1 & 0\\ 0 & 0 & -2 \end{array}\right). \end{array}$$

In view of the procedure described by Equation (13), the Gell–Mann matrices generate the following bosonic operators:

(27) $$\begin{array}{c} {\hat{\lambda }}_{1}={\hat{a}}_{1}^{†}{\hat{a}}_{2}+{\hat{a}}_{2}^{†}{\hat{a}}_{1},\;{\hat{\lambda }}_{2}=i\left({\hat{a}}_{2}^{†}{\hat{a}}_{1}-{\hat{a}}_{1}^{†}{\hat{a}}_{2}\right),\;{\hat{\lambda }}_{3}={\hat{a}}_{1}^{†}a_{1}-{\hat{a}}_{2}^{†}{\hat{a}}_{2},\;{\hat{\lambda }}_{4}={\hat{a}}_{1}^{†}{\hat{a}}_{3}+{\hat{a}}_{3}^{†}{\hat{a}}_{1},\\ {\hat{\lambda }}_{5}=i\left({\hat{a}}_{3}^{†}{\hat{a}}_{1}-{\hat{a}}_{1}^{†}{\hat{a}}_{3}\right),\;{\hat{\lambda }}_{6}={\hat{a}}_{2}^{†}{\hat{a}}_{3}+{\hat{a}}_{3}^{†}{\hat{a}}_{2},\;{\hat{\lambda }}_{7}=i\left({\hat{a}}_{3}^{†}{\hat{a}}_{2}-{\hat{a}}_{2}^{†}{\hat{a}}_{3}\right),\;{\hat{\lambda }}_{8}=\frac{1}{\sqrt{3}}\left({\hat{a}}_{1}^{†}{\hat{a}}_{1}+{\hat{a}}_{2}^{†}{\hat{a}}_{2}-2{\hat{a}}_{3}^{†}{\hat{a}}_{3}\right). \end{array}$$

To obtain the eigenvalues and eigenvectors associated with su(3), we define the following operators:

(28) $$\begin{array}{c} {\hat{T}}_{\pm }=\frac{1}{2}\left({\hat{\lambda }}_{1}\pm i{\hat{\lambda }}_{2}\right),\;{\hat{T}}_{3}=\frac{1}{2}{\hat{\lambda }}_{3},\;{\hat{U}}_{\pm }=\frac{1}{2}\left({\hat{\lambda }}_{6}\pm i{\hat{\lambda }}_{7}\right),\;{\hat{U}}_{3}=\frac{1}{4}\left(-{\hat{\lambda }}_{3}+\sqrt{3}\;{\hat{\lambda }}_{8}\right),\\ {\hat{V}}_{\pm }=\frac{1}{2}\left({\hat{\lambda }}_{4}\pm i{\hat{\lambda }}_{5}\right),\;{\hat{V}}_{3}=\frac{1}{4}\left({\hat{\lambda }}_{3}+\sqrt{3}\;{\hat{\lambda }}_{8}\right), \end{array}$$

where each subgroup $\left\{{\hat{T}}_{\pm },{\hat{T}}_{3}\right\}$, $\left\{{\hat{U}}_{\pm },{\hat{U}}_{3}\right\}$, and $\left\{{\hat{V}}_{\pm },{\hat{V}}_{3}\right\}$ forms a su(2) sub-algebra. These operators can be written in terms of bosonic operators as follows:

(29) $$\begin{array}{c} {\hat{T}}_{+}={\hat{a}}_{1}^{†}{\hat{a}}_{2},\;{\hat{T}}_{-}={\hat{a}}_{2}^{†}{\hat{a}}_{1},\;{\hat{T}}_{3}=\frac{1}{2}\left({\hat{a}}_{1}^{†}{\hat{a}}_{1}-{\hat{a}}_{2}^{†}{\hat{a}}_{2}\right),\\ {\hat{U}}_{+}={\hat{a}}_{2}^{†}{\hat{a}}_{3},\;{\hat{U}}_{-}={\hat{a}}_{3}^{†}{\hat{a}}_{2},\;{\hat{U}}_{3}=\frac{1}{2}\left({\hat{a}}_{2}^{†}{\hat{a}}_{2}-{\hat{a}}_{3}^{†}{\hat{a}}_{3}\right),\\ {\hat{V}}_{+}={\hat{a}}_{1}^{†}{\hat{a}}_{3},\;{\hat{V}}_{-}={\hat{a}}_{3}^{†}{\hat{a}}_{1},\;{\hat{V}}_{3}=\frac{1}{2}\left({\hat{a}}_{1}^{†}{\hat{a}}_{1}-{\hat{a}}_{3}^{†}{\hat{a}}_{3}\right). \end{array}$$

Then, the eigenstates of the system are the eigenstates for the diagonal operators (${\hat{T}}_{3}$, ${\hat{U}}_{3}$, and $V_{3}$), denoted as $|T_{3},V_{3},U_{3}\rangle$; they can also be denoted in the harmonic oscillator representation, namely

(30) $$|T_{3},V_{3},U_{3}\rangle =|n_{1},n_{2},n_{3}\rangle \;\mathrm{for}\;n_{1,2,3}=0,1,\ldots ,\infty .$$

Meanwhile, in this case, the second-order Casimir operator can be obtained as

(31) $${\hat{C}}_{1}=\frac{1}{4}\sum_{j=1}^{8}{\hat{\lambda }}_{j}^{2}=\frac{1}{3}{\left({\hat{n}}_{1}+{\hat{n}}_{2}+{\hat{n}}_{3}\right)}^{2}+\left({\hat{n}}_{1}+{\hat{n}}_{2}+{\hat{n}}_{3}\right),\;\mathrm{with}\;{\hat{n}}_{j}={\hat{a}}_{j}^{†}{\hat{a}}_{j}.$$

These definitions are important in the theory of quantum chromodynamics as these allow us to depict the strong interaction between quarks.

## 3. Bosonic Representation of su(2) Algebra and Applications

In this section, we discuss some properties and advantages of the study of harmonic oscillator coherent states (and its superpositions) under the angular momentum perspective.

The Jordan–Schwinger representation of angular momentum allows us to represent angular momentum states as bimodal bosonic number states. In this representation, the operators forming the Lie algebra are defined as follows:

(32) $${\hat{J}}_{+}={\hat{a}}^{†}\hat{b},\;{\hat{J}}_{-}=\hat{a}{\hat{b}}^{†},\;{\hat{J}}_{z}=\frac{1}{2}\left({\hat{a}}^{†}\hat{a}-{\hat{b}}^{†}\hat{b}\right),$$

where the standard commutation relations are satisfied, $\left[{\hat{J}}_{z},{\hat{J}}_{\pm }\right]=\pm {\hat{J}}_{\pm }$, and $\left[{\hat{J}}_{+},{\hat{J}}_{-}\right]=2{\hat{J}}_{z}$. The eigenvectors of ${\hat{J}}^{2}={\hat{J}}_{+}{\hat{J}}_{-}+{\hat{J}}_{-}{\hat{J}}_{+}+{\hat{J}}_{z}^{2}$ and ${\hat{J}}_{z}$ can be written as number states in the following form:

(33) $$|j,m\rangle =|n,N-n\rangle ,\;\mathrm{with}\;j=\frac{N}{2},\;m=n-\frac{N}{2};$$

here, N=0,1,2,… fixes the value of the total angular momentum, and once this value is fixed, then n=0,1,…,N changes the value of m=−j,−j+1,…,j−1,j. For example, for angular momentum j=0, we have one state with N=0 and n=0, i.e., the vacuum state |0,0〉=|j=0,m=0〉.

For j=1/2, we have N=1 and n=0,1, so we have two states:

(34) |j=1/2,m=−1/2〉=|0,1〉,|j=1/2,m=1/2〉=|1,0〉,

which are states with one total photon.

For j=1, one has that N=2 and n=0,1,2 provide three states

(35) |j=1,m=−1〉=|0,2〉,|j=1,m=0〉=|1,1〉,|j=1,m=1〉=|2,0〉,

which are states with two total quanta. In general, the angular momentum states for *j* correspond to number states with the value of operator ${\hat{a}}^{†}\hat{a}+{\hat{b}}^{†}\hat{b}$ fixed and equal to 2j. In other words, for angular momentum *j*, we have the following 2j+1 states:

(36) |j,m=−j〉=|0,2j〉,|j,m=−j+1〉=|1,2j−1〉,…,|j,m=j−1〉=|2j−1,1〉,|j,m=j〉=|2j,0〉.

From these properties and the completeness of the bimodal harmonic oscillator eigenstates:

$$\sum_{l_{1},l_{2}=0}^{\infty }|l_{1},l_{2}\rangle \langle l_{1},l_{2}|=𝟙,$$

it can be demonstrated that the sum of all the angular momentum states forms a basis of the bimodal harmonic oscillator space:

$$\sum_{j=0}^{\infty }\sum_{m=-j}^{j}\left|j,m\rangle \langle j,m\right|=\sum_{l_{1},l_{2}=0}^{\infty }|l_{1},l_{2}\rangle \langle l_{1},l_{2}|=𝟙,$$

where the angular momentum can take any possible value j=0,1/2,1,3/2,2,… In particular, the number state $|l_{1},l_{2}\rangle$ can be written as the following angular momentum state:

(37) $$|l_{1},l_{2}\rangle =|j=\frac{l_{1}+l_{2}}{2},m=\frac{l_{1}-l_{2}}{2}\rangle .$$

Given this property, symplectic tomograms of the angular momentum states can be obtained, in view of the integral of Equation (10), as follows:

(38) $$\begin{array}{ccc} & & w_{j,m}\left(X,Y|\mu_{1},\nu_{1},\mu_{2},\nu_{2}\right)=\frac{1}{4\pi^{2}}\\ & & \times {\left|\int_{-\infty }^{\infty }\Psi_{l_{1},l_{2}}\left(x_{1},x_{2}\right)\exp\left(\frac{i\mu_{1}}{2\nu_{1}}x_{1}^{2}-\frac{iX}{\nu_{1}}x_{1}+\frac{i\mu_{2}}{2\nu_{2}}x_{2}^{2}-\frac{iY}{\nu_{2}}x_{2}\right)\;dx_{1}\;dx_{2}\right|}^{2}, \end{array}$$

where $\Psi_{l_{1},l_{2}}\left(x_{1},x_{2}\right)=\frac{1}{\pi^{1/2}\sqrt{2^{l_{1}+l_{2}}l1!l2!}}e^{-\frac{1}{2}\left(x_{1}^{2}+x_{2}^{2}\right)}H_{l_{1}}\left(x_{1}\right)H_{l_{2}}\left(x_{2}\right)$ are the bimodal harmonic oscillator eigenfunctions. Thus, the angular momentum tomogram can be written in the following form:

(39) $$\begin{array}{cc} \; & w_{j,m}\left(X,Y|\;\mu_{1},\nu_{1},\mu_{2},\nu_{2}\right)=w_{l_{1}}\left(X|\;\mu_{1},\nu_{1}\right)\;w_{l_{2}}\left(Y|\;\mu_{2},\nu_{2}\right)\\ \; & =\frac{e^{-\frac{X^{2}}{\sqrt{\mu_{1}^{2}+\nu_{1}^{2}}}-\frac{Y^{2}}{\sqrt{\mu_{2}^{2}+\nu_{2}^{2}}}}}{2^{l_{1}+l_{2}}l_{1}!l_{2}!\pi \sqrt{\left(\mu_{1}^{2}+\nu_{1}^{2}\right)\left(\left(\mu_{2}^{2}+\nu_{2}^{2}\right)\right)}}{\left|H_{l_{1}}\left(\frac{X}{\sqrt{\mu_{1}^{2}+\nu_{1}^{2}}}\right)\right|}^{2}{\left|H_{l_{2}}\left(\frac{Y}{\sqrt{\mu_{2}^{2}+\nu_{2}^{2}}}\right)\right|}^{2}\\ \; & =\frac{e^{-\frac{X^{2}}{\sqrt{\mu_{1}^{2}+\nu_{1}^{2}}}-\frac{Y^{2}}{\sqrt{\mu_{2}^{2}+\nu_{2}^{2}}}}}{2^{2j}\left(j+m\right)!\left(j-m\right)!\pi \sqrt{\left(\mu_{1}^{2}+\nu_{1}^{2}\right)\left(\left(\mu_{2}^{2}+\nu_{2}^{2}\right)\right)}}{\left|H_{j+m}\left(\frac{X}{\sqrt{\mu_{1}^{2}+\nu_{1}^{2}}}\right)\right|}^{2}{\left|H_{j-m}\left(\frac{Y}{\sqrt{\mu_{2}^{2}+\nu_{2}^{2}}}\right)\right|}^{2}. \end{array}$$

On the other hand, following the connection between the angular momentum states and the bimodal Fock states of Equation (37), the bimodal coherent state |α,β〉 can also be written as the superposition of Fock states; it reads

(40) $$|\alpha ,\beta \rangle =e^{-\frac{1}{2}{\left(|\alpha \right|}^{2}+{\left|\beta \right|}^{2})}\sum_{l_{1},l_{2}=0}^{\infty }\frac{\alpha^{l_{1}}\beta^{l_{2}}}{\sqrt{l_{1}!l_{2}!}}|l_{1},l_{2}\rangle =e^{-\frac{1}{2}{\left(|\alpha \right|}^{2}+{\left|\beta \right|}^{2})}\sum_{j=0}^{\infty }\sum_{m=-j}^{j}\frac{\alpha^{j+m}\beta^{j-m}}{\sqrt{(j+m)!(j-m)!}}|j,m\rangle .$$

This infinite sum takes into account all different angular momenta. In other words, the coherent state can be represented by an infinite vector in the angular momentum space.

From the coherent state, one can construct different superpositions symmetrical under rotations and/or inversions in the phase space ($p_{1},q_{1},p_{2},q_{2}$). For example, the superpositions associated with the inversion operation in such phase space are

(41) $$|\psi_{2}^{\left(1,2\right)}\rangle =N_{1,2}(|\alpha ,\beta \rangle \pm |-\alpha ,-\beta \rangle ),\;N_{1,2}=\frac{1}{\sqrt{2(1\pm e^{-2(|\alpha {|}^{2}+|\beta {|}^{2})})}};$$

they are also called the even and odd coherent states. These states can be rewritten in the angular momentum representation as

(42) $$|\psi_{2}^{\left(1,2\right)}\rangle =e^{-\frac{1}{2}{\left(|\alpha \right|}^{2}+{\left|\beta \right|}^{2})}N_{1,2}\sum_{j=0}^{\infty }\sum_{m=-j}^{j}\frac{\alpha^{j+m}\beta^{j-m}\left(1\pm {\left(-1\right)}^{2j}\right)}{\sqrt{(j+m)!(j-m)!}}|j,m\rangle ,$$

meaning that the even coherent state has only integer angular momentum contributions, while the odd coherent state has only semi-integer contributions. The other way to say this is that the even state can be represented by an infinite number of boson particles, and the odd state is represented by only fermions. This result is not entirely unexpected and provides the symmetry and antisymmetry of the even and odd coherent states, respectively.

Similarly, other superpositions of coherent states carrying the irreducible representations of cyclic groups of degree *n* can be analyzed in this way. These states have the same rotation symmetries as the *n*-sided regular polygon and can be represented by a superposition of rotations in the phase space. In the case of the cyclic group $C_{3}$, which contains the rotation operations of the regular triangle, we can generate three orthonormal states listed as

(43) $$\begin{array}{c} |\psi_{3}^{\left(1\right)}\rangle =M_{1}(|\alpha ,\beta \rangle +|\mu_{3}\alpha ,\mu_{3}\beta \rangle +|\mu_{3}^{*}\alpha ,\mu_{3}^{*}\beta \rangle ),\\ |\psi_{3}^{\left(2\right)}\rangle =M_{2}(|\alpha ,\beta \rangle +\mu_{3}|\mu_{3}\alpha ,\mu_{3}\beta \rangle +\mu_{3}^{*}|\mu_{3}^{*}\alpha ,\mu_{3}^{*}\beta \rangle ),\\ |\psi_{3}^{\left(3\right)}\rangle =M_{3}(|\alpha ,\beta \rangle +\mu_{3}^{*}|\mu_{3}\alpha ,\mu_{3}\beta \rangle +\mu_{3}|\mu_{3}^{*}\alpha ,\mu_{3}^{*}\beta \rangle ), \end{array}$$

where $\mu_{3}=e^{2\pi i/3}$ is one of the cube roots of the identity, which satisfies the equality $1+\mu_{3}+\mu_{3}^{*}=0$ and the properties $\mu_{3}^{2}=\mu_{3}^{*}$, ${\mu_{3}^{*}}^{2}=\mu_{3}$, with the parameters $M_{1,2,3}$ being normalization constants. To obtain the angular momentum representation of these states, one can use the expression of the coherent states in terms of |j,m〉 given by Equation (40); then, we arrive at the following states:

(44) $$\begin{array}{c} |\psi_{3}^{\left(1\right)}\rangle =e^{-\frac{1}{2}{\left(|\alpha \right|}^{2}+{\left|\beta \right|}^{2})}M_{1}\sum_{j=0}^{\infty }\sum_{m=-j}^{j}\frac{\alpha^{j+m}\beta^{j-m}\left(1+\mu_{3}^{2j}+\mu_{3}^{*2j}\right)}{\sqrt{(j+m)!(j-m)!}}|j,m\rangle ,\\ |\psi_{3}^{\left(2\right)}\rangle =e^{-\frac{1}{2}{\left(|\alpha \right|}^{2}+{\left|\beta \right|}^{2})}M_{2}\sum_{j=0}^{\infty }\sum_{m=-j}^{j}\frac{\alpha^{j+m}\beta^{j-m}\left(1+\mu_{3}^{2j+1}+\mu_{3}^{*2j+1}\right)}{\sqrt{(j+m)!(j-m)!}}|j,m\rangle ,\\ |\psi_{3}^{\left(3\right)}\rangle =e^{-\frac{1}{2}{\left(|\alpha \right|}^{2}+{\left|\beta \right|}^{2})}M_{3}\sum_{j=0}^{\infty }\sum_{m=-j}^{j}\frac{\alpha^{j+m}\beta^{j-m}\left(1+\mu_{3}^{2(j+1)}+\mu_{3}^{*2(j+1)}\right)}{\sqrt{(j+m)!(j-m)!}}|j,m\rangle . \end{array}$$

In view of the properties of the roots of the identity $\mu_{n}=e^{2\pi i/n}$,

(45) $$\sum_{l=1}^{n}\mu_{n}^{l\;r}=e^{\frac{i\pi r(n+1)}{n}}\sin\left(\pi r\right)\csc\left(\frac{\pi r}{n}\right),$$

for n=3; then, the previous formula reads

(46) $$\begin{array}{cc} \sum_{l=1}^{3}\mu_{3}^{l\;(2j)}=3\;\mathrm{if}\;\mathrm{mod}\left(2j,3\right)=0, & \sum_{l=1}^{3}\mu_{3}^{l\;(2j+1)}=3\;\mathrm{if}\;\mathrm{mod}\left(2j+1,3\right)=0,\\ \; & \sum_{l=1}^{3}\mu_{3}^{l\;2(j+1)}=3\;\mathrm{if}\;\mathrm{mod}\left(2\left(j+1\right),3\right)=0, \end{array}$$

while, for any other value of *j*, such sums are equal to zero. These expressions allow us to rewrite the states associated with the $C_{3}$ group as follows:

(47) $$\begin{array}{c} |\psi_{3}^{\left(1\right)}\rangle =3M_{1}^{\prime }\sum_{j=0,\frac{3}{2},3,\frac{9}{2},6,\frac{15}{2},9,\cdots }\sum_{m=-j}^{j}\frac{\alpha^{j+m}\beta^{j-m}}{\sqrt{(j+m)!(j-m)!}}|j,m\rangle ,\\ |\psi_{3}^{\left(2\right)}\rangle =3M_{2}^{\prime }\sum_{j=1,\frac{5}{2},4,\frac{11}{2},7,\frac{17}{2},10,\cdots }\sum_{m=-j}^{j}\frac{\alpha^{j+m}\beta^{j-m}}{\sqrt{(j+m)!(j-m)!}}|j,m\rangle ,\\ |\psi_{3}^{\left(3\right)}\rangle =3M_{3}^{\prime }\sum_{j=\frac{1}{2},2,\frac{7}{2},5,\frac{13}{2},8,\frac{19}{2},11,\cdots }\sum_{m=-j}^{j}\frac{\alpha^{j+m}\beta^{j-m}}{\sqrt{(j+m)!(j-m)!}}|j,m\rangle , \end{array}$$

where $M_{i}^{\prime }={\exp[-(|\alpha |}^{2}+{\left|\beta \right|}^{2})/2]M_{i}$.

As an example, we explicitly write the cyclic states for α=1/10 and β=i/10. In this case, the cyclic states can be obtained, in view of Equation (47), resulting in the following:

(48) $$\begin{array}{c} |\psi_{3}^{\left(1\right)}\rangle =M_{1}^{\prime }\left(3|0,0\rangle +\frac{1}{1000}\sqrt{\frac{3}{2}}\left(|\frac{3}{2},\frac{3}{2}\rangle +i\sqrt{3}|\frac{3}{2},\frac{1}{2}\rangle -\sqrt{3}|\frac{3}{2},-\frac{1}{2}\rangle -i|\frac{3}{2},-\frac{3}{2}\rangle \right)+O\left(10^{-6}\right)\right),\\ |\psi_{3}^{\left(2\right)}\rangle =M_{2}^{\prime }\left(\frac{3}{100}\left(\frac{1}{\sqrt{2}}|1,1\rangle +i|1,0\rangle -\frac{1}{\sqrt{2}}|1,-1\rangle \right)+O\left(10^{-6}\right)\right),\\ |\psi_{3}^{\left(3\right)}\rangle =M_{3}^{\prime }(\frac{3}{10}\left(|\frac{1}{2},\frac{1}{2}\rangle +i|\frac{1}{2},-\frac{1}{2}\rangle \right)+\frac{1}{10000}\sqrt{\frac{3}{2}}(\frac{1}{2}|2,2\rangle +i|2,1\rangle -\sqrt{\frac{3}{2}}|2,0\rangle \\ -i|2,-1\rangle +\frac{1}{2}|2,-2\rangle )+O\left(10^{-6}\right)); \end{array}$$

they form an orthogonal set of states.

In general, a set of *n* orthonormal states, associated with the $C_{n}$ group, can be generated, using the following expression:

(49) $$|\psi_{n}^{\left(\lambda \right)}\rangle =M_{n}^{\left(\lambda \right)}\sum_{k=1}^{n}\chi_{\lambda ,r}^{\left(n\right)}|\alpha_{r},\beta_{r}\rangle ,$$

where λ=1,…,n enumerates the number of states, and $\chi_{\lambda ,r}^{\left(n\right)}$ is the character for the *r*-th group element of the λ-th irreducible representation of the cyclic group $C_{n}$. In other words, $\chi_{\lambda ,r}^{\left(n\right)}=\mu_{n}^{\left(\lambda -1)(r-1\right)}$ are roots of unity, and $\alpha_{r},\beta_{r}=\mu_{n}^{r-1}\alpha ,\mu_{n}^{r-1}\beta$, which result from the application of the *r*-th element of $C_{n}$ on the complex numbers α and β, respectively. The normalization constant can be determined by the following expression:

(50) $${\left(M_{n}^{\left(\lambda \right)}\right)}^{-2}=\sum_{s,s^{\prime }=1}^{n}\chi_{\lambda ,s}^{\left(n\right)}\;\chi_{\lambda ,s^{\prime }}^{(n)*}e^{-\frac{1}{2}\left(\right|\alpha_{s}{|}^{2}+|\alpha_{s^{\prime }}{|}^{2}+|\beta_{s}{|}^{2}+|\beta_{s^{\prime }}{|}^{2})+\alpha_{s}\alpha_{s^{\prime }}^{*}+\beta_{s}\beta_{s^{\prime }}^{*}}.$$

This general expression can be used to write any state associated with $C_{n}$. Using Equations (40) and (49), one can write the state corresponding to the λ-th irreducible representation of $C_{n}$ in angular momentum form as follows:

(51) $$|\psi_{n}^{\left(\lambda \right)}\rangle =M_{n}^{\left(\lambda \right)}\sum_{r=1}^{n}\chi_{\lambda ,r}^{\left(n\right)}\sum_{j=0}^{\infty }\sum_{m=-j}^{j}\frac{\alpha_{r}^{j+m}\beta_{r}^{j-m}e^{-\frac{1}{2}\left(\right|\alpha_{r}{|}^{2}+|\beta_{r}{|}^{2})}}{\sqrt{(j+m)!(j-m)!}}|j,m\rangle .$$

This equation allow us to study the properties of the cyclic states in the angular momentum representation. For example, one can see the properties of a Husimi-like *Q* representation or the symplectic tomographic representation in terms of angular momentum variables.

## 4. Probabilistic Representation of Cyclic States

The extension of pseudo-probability distributions for any Lie group is a nontrivial procedure [48,49], but we take advantage of the fact that the use of the Husimi function has been previously extended to the su(2) algebra [44]. In this section, we use this knowledge to present a possible generation of the su(2) Husimi function for the case of a superposition of an infinite number of angular momenta. This Husimi-like function together with the symplectic tomogram of the cyclic states are used to visualize and characterize the cyclic states.

The cyclic states mentioned in the previous section can be studied in terms of the su(2) coherent states. We recall the expression of such coherent states $|\xi ,j\rangle =\exp\left(\xi {\hat{J}}_{+}-\xi^{*}{\hat{J}}_{-}\right)|j,-j\rangle$ in terms of the eigenstates of ${\hat{J}}^{2}$ and ${\hat{J}}_{z}$, namely

(52) $$|\xi ,j\rangle =\frac{1}{{\left(1+|\nu \right|}^{2}{)}^{j}}\sum_{m=-j}^{j}{\left(\frac{(2j)!}{(j+m)!(j-m)!}\right)}^{1/2}\nu^{j+m}|j,m\rangle ,\;\mathrm{with}\;\nu =\frac{\xi }{\left|\xi \right|}\tan\left|\xi \right|.$$

The coherent states in the previous expression are sometimes only referred to as |ν,j〉 and form an overcomplete basis for the states with a fixed *j*. That is, the unity operator can be written as

(53) $$𝟙=\left(2j+1\right)\int \frac{\left|\nu ,j\rangle \langle \nu ,j\right|}{{\pi (1+|\nu |}^{2}{)}^{2}}\;d^{2}\nu ,$$

for any j=0,1/2,1,… These states lead us to the following overlap between the coherent states and |j,m〉:

(54) $$\langle \nu ,j|j,m\rangle =\frac{1}{{\left(1+|\nu \right|}^{2}{)}^{j}}{\left(\frac{(2j)!}{(j+m)!(j-m)!}\right)}^{1/2}\nu^{*(j+m)}.$$

Defining this scalar product for a fixed angular momentum and its projection as $\langle \nu ,j|j,m\rangle =f_{j,m}$ and assuming a general state constructed from every angular momentum contribution:

(55) $$|\psi \rangle =\sum_{j=0}^{\infty }\sum_{m=-j}^{j}C_{j,m}\;|j,m\rangle ,\;\mathrm{with}\;\sum_{j=0}^{\infty }\sum_{m=-j}^{j}{\left|C_{j,m}\right|}^{2}=1,$$

one can define the following function:

(56) $$Q_{|\psi \rangle }\left(\nu \right)=\frac{2j+1}{{\pi (1+|\nu |}^{2}{)}^{2}}\sum_{j=0}^{\infty }\sum_{m=-j}^{j}|C_{j,m}{|}^{2}\;{\left|f_{j,m}\right|}^{2},$$

which is the convex sum of the scalar product 〈ν,j|j,m〉 for different *j* and *m*. It can be demonstrated that $Q_{|\psi \rangle }\left(\nu \right)$ is a normalized function in the complex space associated with the coherent state parameter ν, i.e.,

(57) $$\int Q_{|\psi \rangle }\left(\nu \right)\;d^{2}\nu =1.$$

To show this property, one can substitute Equation (54) into Equation (56) and use the following integral:

(58) $$\int \frac{{\left|\nu \right|}^{2(j+m)}}{{\left(1+|\nu \right|}^{2}{)}^{2(j+1)}}\;d^{2}\nu =\frac{\pi (j+m)!(j-m)!}{(2j+1)!}.$$

Thus, we arrive at the following expression:

(59) $$\int Q_{|\psi \rangle }\left(\nu \right)\;d^{2}\nu =\sum_{j=0}^{\infty }\sum_{m=-j}^{j}{\left|C_{j,m}\right|}^{2}=1.$$

Given that the function $Q_{|\psi \rangle }\left(\nu \right)$ is normalized and positive semi-definite and contains information on the state |ψ〉 and the coherent states |ν,j〉, then we call it the Husimi-like function and study some of its properties for the harmonic oscillator coherent states |α,β〉 and the cyclic states $|\psi_{n}^{\left(\lambda \right)}\rangle$.

The Husimi-like function is used to analyze the two-mode coherent state of Equation (40) and the cyclic states of Equations (41) and (44). The coherent states, in the angular momentum representation, read

(60) $$\langle \nu ,j|\alpha ,\beta \rangle =\frac{\alpha^{j+m}\beta^{j-m}e^{-\frac{1}{2}{\left(|\alpha \right|}^{2}+{\left|\beta \right|}^{2})}}{(j+m)!(j-m)!}\frac{\sqrt{(2j)!}}{{\left(1+|\nu \right|}^{2}{)}^{j}}\nu^{*(j+m)};$$

with this expression and the definition for the Husimi-like probability distribution (Equation (56)), one can obtain $Q_{|\alpha ,\beta \rangle }\left(\nu \right)$ for the bimodal light coherent state |α,β〉 in the su(2) coherent state |ν,j〉 representation. This probability distribution is defined as

(61) $$\begin{array}{ccc} Q_{|\alpha ,\beta \rangle }\left(\nu \right) & = & \sum_{j=0}^{\infty }\sum_{m=-j}^{j}\frac{{\left(2j+1\right)|\langle \nu ,j|\alpha ,\beta \rangle |}^{2}}{{\pi (1+|\nu |}^{2}{)}^{2}}\\ \; & = & \sum_{j=0}^{\infty }\sum_{m=-j}^{j}\frac{{\left(2j+1\right)!|\alpha |}^{2(j+m)}{\left|\beta \right|}^{2(j-m)}{\left|\nu \right|}^{2(j+m)}e^{-(|\alpha {|}^{2}+|\beta {|}^{2})}}{\pi {\left(\left(j+m\right)!\left(j-m\right)!\right)}^{2}{\left(1+|\nu \right|}^{2}{)}^{2(j+1)}}. \end{array}$$

This function is normalized and provides the sum of overlap probabilities of the su(2) coherent state for different angular momenta *j* and our harmonic-oscillator coherent state |α,β〉.

Analogous to the Husimi-like function for the coherent state, the Husimi-like probability distribution associated with the cyclic states of Equation (51) can be obtained. The resulting probability distribution $Q_{|\psi_{n}^{\left(\lambda \right)}\rangle }\left(\nu \right)$ has the form

(62) $$\begin{array}{c} Q_{|\psi_{n}^{\left(\lambda \right)}\rangle }\left(\nu \right)={\left(M_{n}^{\left(\lambda \right)}\right)}^{2}\sum_{j=0}^{\infty }\;\sum_{m=-j}^{j}\;\sum_{r,r^{\prime }=1}^{n}\chi_{\lambda ,r}^{\left(n\right)}\chi_{\lambda ,r^{\prime }}^{(n)*}\frac{\left(2j+1\right)!{\left(\alpha_{r}\alpha_{r^{\prime }}^{*}\right)}^{j+m}{\left(\beta_{r}\beta_{r^{\prime }}^{*}\right)}^{j-m}{\left|\nu \right|}^{2(j+m)}e^{-(|\alpha {|}^{2}+|\beta {|}^{2})}}{\pi {\left(\left(j+m\right)!\left(j-m\right)!\right)}^{2}{\left(1+|\nu \right|}^{2}{)}^{2(j+1)}}, \end{array}$$

where we emphasize that $\chi_{\lambda ,r}^{\left(n\right)}=\mu_{n}^{\left(\lambda -1)(r-1\right)}$, $\alpha_{r},\beta_{r}=\mu_{n}^{r-1}\alpha ,\mu_{n}^{r-1}\beta$, and $\mu_{n}=e^{2\pi i/n}$. The probability distributions of Equations (61) and (62) allow us to visualize the two-mode coherent states and the cyclic state in terms of the real and imaginary parts or the polar coordinates of the su(2) coherent-state parameter $\nu =\nu_{R}+i\nu_{I}=\rho e^{i\phi }$. Since this parameter ν is characterized only by two parameters (the phase ϕ and norm ρ) and sometimes only one parameter (the norm of ν), the Husimi-like function Q(ν) allows us to visualize the two-mode states |α,β〉 or its superpositions; this is an advantage, since, generally, we cannot provide this visualization, using the standard *Q* representation with optical coherent states, i.e., $Q\propto {\left|\langle \gamma ,\delta |\alpha ,\beta \rangle \right|}^{2}$, as the latter requires four parameters, making the representation four-dimensional.

**Figure 1:**
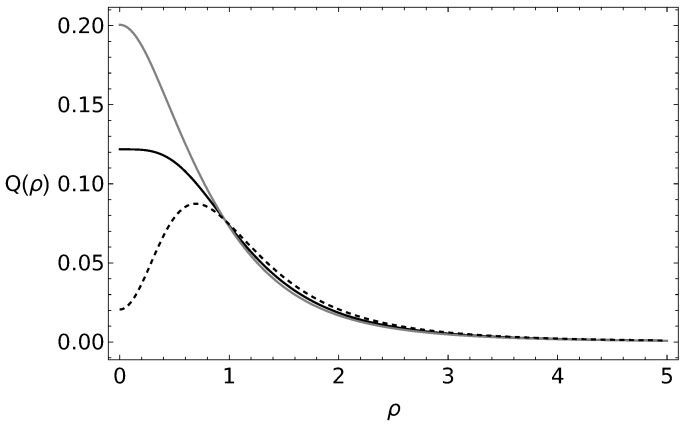
Husimi-like Q(ν) probability distribution for the coherent state (black) and the cyclic states, even (gray) and odd (black, dashed), associated with the $C_{2}$ group. Here, the parameters α=1, β=i/5, and $\nu =\rho e^{i\phi }$.

**Figure 2:**
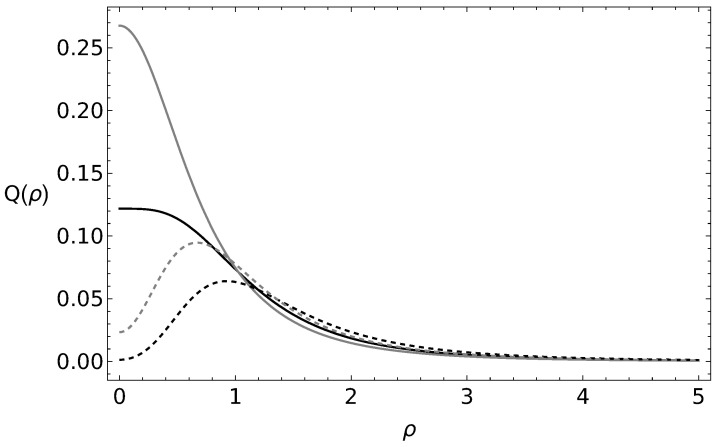
Husimi-like Q(ν) probability distribution for the coherent state (black), cyclic states with irreducible representation λ=1 (gray), λ=2 (black, dashed), and λ=3 (gray, dashed), associated with the $C_{3}$ group. Here, the parameters α=1, β=i/5, and $\nu =\rho e^{i\phi }$.

In Figure 1, we show the Husimi-like *Q* probability distribution for the coherent states and both cyclic states for the $C_{2}$ group for α=1 and β=i/5. The plot is presented in terms of the norm of the su(2) coherent states $\nu =\rho e^{i\phi }$. One can see the difference between the *Q* representation for all the states. In Figure 2, we present the probability distribution for the cyclic states associated with the $C_{3}$ group for the same coherent parameters α=1 and β=i/5. The probability distributions for λ=2 and λ=3 present a similar behavior, although they can be discerned between each other.

We point out again that Equations (61) and (62) satisfy the normalization condition:

(63) $$\int Q_{|\psi \rangle }\left(\nu \right)\;d^{2}\nu =1,$$

which can be checked, using the integral Equation (58) together with Equation (50). These normalization conditions result from the normalization of the coherent state and cyclic states and due to the fact that the definition of our Husimi-like function (56) represents a convex sum of different angular momentum contributions.

In addition to the Husimi-like function, one can inspect the symplectic tomogram of the cyclic states. The symplectic tomographic representation of the cyclic states can be obtained using the position representation of the bimodal coherent states |α,β〉:

(64) $$\Psi \left(x_{1},x_{2}\right)=\frac{1}{\pi^{1/2}}\exp\left(-\frac{x_{1}^{2}}{2}-\frac{x_{2}^{2}}{2}+\sqrt{2}\alpha x_{1}+\sqrt{2}\beta x_{2}-\frac{1}{2}(\alpha^{2}+{\left|\alpha \right|}^{2})-\frac{1}{2}(\beta^{2}+{\left|\beta \right|}^{2})\right).$$

Employing the definition of symplectic tomogram, we arrive at the following sum:

(65) $$\begin{array}{ccc} \; & \; & w_{|\psi_{r}^{\left(\lambda \right)}\rangle }\left(X,Y|\;\mu_{1},\nu_{1},\mu_{2},\nu_{2}\right)=\frac{1}{\pi }\frac{1}{\sqrt{\left(\mu_{1}^{2}+\nu_{1}^{2}\right)\left(\mu_{2}^{2}+\nu_{2}^{2}\right)}}\\ \; & \; & \times {\left|M_{\lambda }^{\left(n\right)}\sum_{r=1}^{n}\chi_{\lambda ,r}^{\left(n\right)}e^{-\frac{X^{2}}{2\nu_{1}\left(\nu_{1}-i\mu_{1}\right)}+\frac{\sqrt{2}\alpha_{r}X}{\mu_{1}+i\nu_{1}}-\frac{1}{2}(\alpha_{r}^{2}+|\alpha_{r}{|}^{2})}e^{-\frac{Y^{2}}{2\nu_{2}\left(\nu_{2}-i\mu_{2}\right)}+\frac{\sqrt{2}\beta_{r}Y}{\mu_{2}+i\nu_{2}}-\frac{1}{2}(\beta_{r}^{2}+|\beta_{r}{|}^{2})+i\left(\frac{\alpha_{r}^{2}\nu_{1}}{\mu_{1}+i\nu_{1}}+\frac{\beta_{r}^{2}\nu_{2}}{\mu_{2}+i\nu_{2}}\right)}\right|}^{2}. \end{array}$$

**Figure 3:**
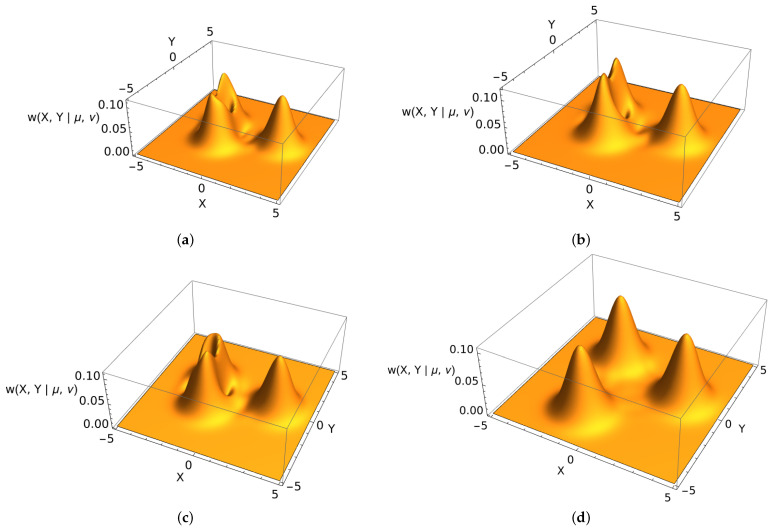
Tomographic representation for the cyclic states associated with the cyclic group $C_{3}$ with irreducible representations λ=1,2,3 (**a**–**c**, respectively) associated with the $C_{3}$ group; here, the parameters α=2 and β=i. Tomographic representation for λ=1, α=2, β=2i (**d**). For all the cases, the parameters $\mu_{1}=\cos\left(1/10\right)$, $\nu_{1}=\sin\left(1/10\right)$, $\mu_{2}=\cos\left(1/5\right)$, and $\nu_{2}=\sin\left(1/5\right)$.

In Figure 3, we present the tomographic representation for the cyclic states associated with the group $C_{3}$ for all possible irreducible representations $\lambda_{1,2,3}$. We can see that all the states have different tomographic probability distributions, when the coherent parameters α and β have different absolute values |α|≠|β|. In Figure 3, we also show that, in the case where both absolute values are equal |α|=|β|, the original symmetry of the states arises also in the tomographic representation. As in the case of the Husimi-like probabilistic representation, the tomogram can be used to have a graphic representation of the state.

As the cyclic states form a system of orthonormal states, then one can use them as a basis to encode a qudit quantum system. As we see, we have three cyclic states $|\psi_{n}^{\left(\lambda \right)}\rangle$ for λ=1,2,3, which can encode a qutrit system. Similar to the Gottesman–Kitaev–Preskill (GKP) code [50], here, we have continuous quantum variable systems, which can reduce certain types of noise.

## 5. Summary and Concluding Remarks

Based on the Jordan–Schwinger representation, we defined a general procedure to obtain the bosonic representation of a group of matrices with their given commutation relations. We explicitly obtained the cases of the su(2) and su(3) algebras and discussed some of their properties. In the su(2) case, the bosonic representation was used to link the angular momentum states with the bimodal Fock number states. Later, we used this property to characterize bimodal optical coherent states and their superpositions with angular momentum sums. In the case of the cyclic states, an explicit expression for its description in terms of angular momentum states is given in Equation (51). We point out that, in the case of the even and odd coherent states, we have superpositions of only bosonic and fermionic states, respectively.

We used the correspondence between angular momentum states and Fock number states to obtain the symplectic tomographic probability distribution representation of angular momentum states in terms of standard Hermite polynomials (Equation (39)) and to calculate a Husimi-like probability distribution of optical coherent and cyclic states (Equations (61) and (62)) making use of the su(2) coherent states. As examples, we showed the probability distribution for the three cyclic states associated with the $C_{3}$ group, which contained the symmetries of the equilateral triangle.

As the cyclic states define an orthonormal set of states, they are suitable to be used in a quantum information context; on the other hand, the correspondence between angular moment states and coherent and cyclic states may allow the simulation of optical systems, using quantum devices based on the angular momentum of different particles. For this, the superposition of momentum states should be considered.

Additionally, the probability distributions constructed in this work, describing the quantum states (Schrödinger cat states), which are superpositions of Fock states, have specific new properties, namely they describe entangled quantum states. Due to this fact, they are entangled probability distributions, which were not studied in classical probability theory. The specific properties of these probability distributions, including inequalities characterizing the Shannon entropy of these entangled distributions, will be studied in a future publication.

## Data Availability

Data sharing is not applicable to this article.
